# Assessing food appeal and desire to eat: the effects of portion size & energy density

**DOI:** 10.1186/1479-5868-8-101

**Published:** 2011-09-25

**Authors:** Kyle S Burger, Marc A Cornier, Jan Ingebrigtsen, Susan L Johnson

**Affiliations:** 1 Department of Pediatrics, Section of Nutrition, University of Colorado Denver, Aurora, CO, USA; 2 The Department of Food Science and Human Nutrition, Colorado State University, Fort Collins, CO, USA; 3 Division of Endocrinology, Metabolism, and Diabetes, Department of Medicine, University of Colorado Denver, Aurora, CO, USA; 4 Center for Human Nutrition, University of Colorado Denver, Denver, CO, USA

**Keywords:** liking, wanting, food appeal, desire to eat, intake, hedonic, obesity, portion size

## Abstract

**Background:**

Visual presentation of food provides considerable information such as its potential for palatability and availability, both of which can impact eating behavior.

**Methods:**

We investigated the subjective ratings for food appeal and desire to eat when exposed to food pictures in a fed sample (n = 129) using the computer paradigm ImageRate. Food appeal and desire to eat were analyzed for the effects of food group, portion size and energy density of the foods presented as well as by participant characteristics.

**Results:**

Food appeal ratings were significantly higher than those for desire to eat (57.9 ± 11.6 v. 44.7 ± 18.0; *p *< 0.05). Body mass index was positively correlated to desire to eat (*r *= 0.20; *p *< 0.05), but not food appeal. Food category analyses revealed that fruit was the highest rated food category for both appeal and desire, followed by discretionary foods. Additionally, overweight individuals reported higher ratings of desire to eat large portions of food compared to smaller portions (*p *< 0.001), although these effects were relatively small. Energy density of the foods was inversely correlated with ratings for both appeal and desire (*r*'s = - 0.27; *p*'s < 0.01).

**Conclusions:**

Results support the hypothesis that individuals differentiate between food appeal and desire to eat foods when assessing these ratings using the same type of metric. Additionally, relations among food appeal and desire to eat ratings and body mass show overweight individuals could be more responsive to visual foods cues in a manner that contributes to obesity.

## Background

Food intake is influenced by a number of factors such as visual food cues in the eating environment, the hedonic value of food and an individual's energy state [1-3]. In today's environment individuals are presented with visual food cues on a continual basis. Images of foods appear in print media, on screen and are visually presented when others are eating. By simply seeing food one is aware of its availability and potential palatability, both of which can act as incentive to initiate food intake [4]. Studies have reported that altering the visual aspects of food, such as portion size and visibility, can increase food intake [5-7], yet little is known about the mechanisms by which this occurs. To understand the possible physiologic basis of the effect of visual presentation of food, research has assessed brain activation in response to food pictures. These studies have reported that brain activation in reward and attention related areas is increased when individuals are shown pictures of energy dense, highly palatable foods [8] and that activation resulting from high calorie foods is positively associated with body mass index [8-10]. However little is known about individuals' preferences for these types of food items and their interactions with food characteristics known to influence food selection (e.g., food categories, portion size, and energy density) individual characteristics such as body mass and levels of dietary restraint and disinhibition.

### Food Liking & Wanting

A positive hedonic value of food, or food reward, is a powerful determinant of eating behavior [11]. Neural responses to positive hedonic food cues occur on two levels, 'liking' and 'wanting' and have independent neural pathways [12]. While liking is commonly conceptualized as the positive hedonic value of food and frequently measured via visual analog scale, wanting referred to the incentive salience and/or motivation to consume that food item [12,13] and has been measured in a variety of ways [14-18]. Epstein and colleagues interpreted Berridge's "motivation to consume" as the amount of work (e.g., pushing a button) an individual would perform to receive food [14]. Wanting has also been measured by asking a participant to choose a preferred food (between two food items) and measuring reaction time [17]. Finlayson and colleagues selected this 'forced choice' method citing that individuals may not be able to differentiate between subjective liking and wanting and that wanting could occur unconsciously [17], although this has not been empirically tested. Whereas in a repeated measure experiment, Liem and colleagues (2009) used a Lykert type scale after children tasted foods [18]. Further, there are few empirical data available that test the ability to reliably assess differences in subjective liking and wanting in a similar metric in adults (for example, visual analog scales). However, based on the differences in these methodologies and lack of the ability to test against a gold-standard, it is unclear if they are measuring the same specific construct. Additionally few data are available regarding the relations between liking and wanting and hunger and satiation. Epstein and colleagues have shown that the depravation of food can selectively influence the willingness to work for food [14]. It has been hypothesized that the physiological state of hunger can influence desire to eat [19], but it also has been reported that subjective food liking operates independently of perceived hunger [20].

### The Eating Environment & Portion Size

The effect of large portions on food intake is well researched. The influence and change in portion sizes over the past three decades have been suggested to significantly contribute to the obesity epidemic [21]. Portion sizes have increased in a near parallel rate to the rise in obesity [22] and increases in portion sizes have been shown to markedly increase food and energy intake in both children [23,24] and adults [5,25,26], although only few studies have reported a direct relationship between portions of food and BMI [7,27,28]. Currently there is a gap in the literature regarding the mechanisms by which portion size impacts intake. We previously theorized that the initial amount of food presented could impact food intake based on reports that removing the visual cue of food after seeing the meal (via blindfolding) did not attenuate the portion size effect [7]. To date, few studies have directly assessed food appeal and desire to eat of a variety of foods varied by portion size without the confound effects of satiety.

### Study Aims and Hypotheses

This study aimed to assess the reliability of a computer paradigm and image set that could be easily disseminated to investigate individuals' characteristics and similarities and differences between food appeal and desire to eat. In addition, we aimed to examine the relationships among food appeal ratings and ratings of desire to eat, food categories, portion size, energy density, and participant characteristics. Our primary hypotheses included: 1) food appeal and desire to eat would be positively correlated, but individuals would differentiate between these two constructs, specifically desire to eat would be rated lower by nature of the controlled full energy state of the participants (see methods); 2) when separating food images by category, discretionary foods (desserts, energy dense foods) would be rated highest in both food appeal and desire to eat; 3) BMI, hunger and dietary disinhibition would be positively associated with desire and appeal ratings, whereas fullness and dietary restraint would have inverse relations with desire and appeal ratings; 4) individuals would rate large portions of food higher for appeal and desire; and 5) energy density of food would be positively associated with food appeal and desire to eat ratings.

## Methods

### Image Set Development

Over 600 images of food were considered for inclusion into the set including those: 1) taken by lab personnel; 2) obtained via the internet; 3) from the International Affective Picture System [29]; 4) and used in previous fMRI research [30,31]. Permission was obtained to use images downloaded from the internet and from previous research. All images were matched for brightness and contrast using Microsoft Office Picture Manager^® ^(Microsoft, Seattle WA, 2007), were sized to be at least 800 × 600 pixels and were converted to a JPEG file type. After the images were standardized, images were excluded if: the image quality (clarity, brightness, contrast) was poor or the food could not be easily identified; if there was an overrepresentation of a kind of food item; or if the food was presented in a manner not typical for consumption (e.g., a whole, uncut pineapple). Liquids were excluded from the image set due to the difficulty in identifying the liquid. Images were selected to represent a variety of ethnic foods and food categories as well as different portion sizes.

Food images were assigned to categories similar to food groups presented in the Health and Human Services 2005 Dietary Guidelines for Americans [32]. When a mixed plate of food was shown, the category assigned represented the predominant food item in the image, as determined by five members of the research staff. If there was no predominant food, the image was placed into a "mixed dish" category. Examples of foods represented in each category and the number of images in each category can be seen in Table 1.

**Table 1:** Descriptive information of the food images and categories selected for the ImageRate program

	Number of Images Included	Examples of Foods in Each Category
Fruit	18	Strawberries, ready to eat oranges and mixed fruit platters
Discretionary foods	21	Brownies, ice cream, cakes and high calorie savory foods such as French fries, and potato chips.
Grains	16	Breads, pastas, bagels and cereals
Dairy	5	Different types of cheese and butter
Vegetables	15	Broccoli, baked potatoes, peas and mixed vegetable dishes (e.g., salad or salsa)
Mixed dishes	28	A plate with eggs and hash browns, a basket of fish and chips and pizza with meat and vegetable toppings
Protein	24	A steak, chicken, seafood, and eggs

Total	127	

A total 165 food images were presented to each participant in a random order. This included 104 unique food images, 46 images of foods which varied by portion size (23 food pairs) and 15 repeated images for reliability analyses.

The 46 portion size images were all photographed by research staff. Twenty-three foods were presented in a small portion (based on one serving per manufacturer nutritional facts label or USDA guidelines) and a large portion (double the small portion). Each of the portion size images was photographed with identical presentation on the same plate in the same lighting. The angle and distance from which these images were taken were based on how an average height male (5' 10") would see a food if seated at a table. These techniques were used to ensure the scale of the foods included in the portion size images consistent and apparent.

For reliability analyses, five sets of 15 images were randomly selected from the original 150. One of the five sets was imbedded randomly into the original set of 150 images for each participant. Thus, each participant rated a total of 165 images in their session. Across the entire sample of participants, reliability data was collected on a total of 75 different images (5 sets of 15 images). Reliability was not assessed on all 150 images to minimize participant burden.

### ImageRate Computer Paradigm

The computer program ImageRate was written in Visual Basic for Applications (Microsoft, Seattle WA, 2007) and Microsoft Office Access^® ^(Microsoft, Seattle WA, 2007) was used as user interface and data storage. The images were presented to the participant one at a time in a random order on a 17 inch (176 cm) monitor. Ratings were assessed for each image by visual analog scales (VAS; 0-100) measuring food appeal and desire to eat the food presented in the image. The question measuring food appeal was phrased 'How appealing is this food?' anchored by 'Not appealing at all' to 'Extremely appealing.' Appeal was defined as the amount the person liked the presented food item. The question to measuring desire to eat was phrased, 'How much do you desire to eat this food?' anchored by 'I have no desire to eat this food' to 'I have a strong desire to eat this food.' Desire to eat was specifically defined as the drive to consume some of the presented food at that point in time. The VAS were presented under the image of the food one at a time and participants progressed through the images and questions at their own pace.

### Measures & Procedures

A total of 130 individuals (M = 56, F = 74) completed the study. One woman was excluded from analysis due to an outlying BMI (62.4). This participant's BMI was four SD above the mean and a Cook's Distance greater than 1 revealed that her BMI was an overly influential data point. All subsequent analyses are presented on the remaining 129 participants. Seventy-four participants (M = 27, F = 47) were classified as lean (BMI 21.6 ± 1.9) and 55 participants (M = 29, F = 26) were classified as overweight (BMI 31.7 ± 6.8). Demographic information and participant characteristics are presented in Table 2. Participants were recruited via flyer, email distribution lists and website message boards regarding a study investigating 'opinions about food' in the Denver Metro and Northern Colorado areas. No further information regarding the purpose of the study was given. Individuals were excluded if they had a visual disability that would affect the ability to differentiate colors or impair seeing in the dark or any developmental disability that could impact data collection.

**Table 2:** Sample description and characteristics

	Men	Women	Total
n	56	73	129
Age (y)	34.5 (11.2)	33.3 (11.3)	33.8 (11.5)
BMI	26.0 (5.5)	25.7 (7.8)	25.9 (6.8)
Education (y)	16.1 (1.2)^a^	15.5 (1.7)^b^	15.7 (1.5)
Dietary Restraint^#^	8.1 (4.5)	9.0 (4.5)	8.6 (4.5)
Dietary Disinhibition^#^	5.3 (3.3)	6.4 (3.7)	5.9 (3.6)
Hunger	35.0 (23.3)^a^	25.2 (21.3)^b^	29.5 (22.6
Fullness	45.6 (23.9)^a^	59.0 (22.1)^b^	53.2 (23.7)

Values are presented in mean (SD)

^a, b ^Different superscripts indicate significant differences between men and women *p *< 0.05

^# ^Measured via the Three Factor Eating Questionnaire [33]. Scale ranges: restraint (0 - low reported restraint, 21 - high dietary restraint); disinhibition (0 - low reported disinhibition, 16 -high reported disinhibition)

Each participant attended one session conducted either at the University of Colorado -Denver or Colorado State University. Prior to the session participants were asked to adhere to their normal eating habits, upon arrival, once informed consent was reviewed and obtained, participants were first asked to drink ≥ 80% of a Boost^® ^nutritional drink (Nestlé HealthCare Nutrition, Fremont, MI 2008) to control for individuals' hunger/fullness levels across the sample. The nutritional drink contained 240 kcal, 10 grams of protein and 4 grams of fat. A gap of 15 minutes was placed between consumption of the nutritional drink and ratings to allow for the satiating effect of the supplement to occur. Participants were then asked to fill out VAS for hunger and fullness (0-100; ranging from not hungry/full at all to extremely hungry/full) prior to the ImageRate procedure.

The participant was given instructions on how to use the ImageRate program and given the opportunity to practice with the assistance of the researcher to ensure complete understanding of the procedures. All ratings were completed on the same 17 inch (176 cm) computer monitor in a quiet, dimly lit, private room. Once the image rating was finished, the participant then completed the Three Factor Eating Questionnaire (TFEQ) [33]. The TFEQ assesses dietary behaviors designed to produce weight loss or maintenance, monitoring of body shape, and importance of thinness (sample item: *I count calories as a conscious means of controlling my weight*). This scale has shown internal consistency (α's ranged from .85 to .93) and temporal reliability; 1-month test-retest *r *= .98 [33,34]. Constructs of interest for this investigation were dietary restraint (range 0-21) and dietary disinhibition (range 0-16). At the end of the session, the participant's height and weight were measured with a standardized scale and stadiometer. All procedures and measures were approved by the Colorado Multiple Institutional Review Board.

### Statistical Analyses

Statistical analyses were performed using SAS Version 9.1 (SAS Institute Inc, Cary, NC 2003). All tests were two-sided, with the significance level set at *p *< 0.05. Data are presented as mean ± standard deviation (SD) unless otherwise specified. Descriptive statistics were performed on all data including means, SD, standard error of the means (SEM) and weight status. Weight status was determined by calculating body mass index (BMI; kg/m^2^) and then BMIs were dichotomized into lean (BMI < 25) and overweight (BMI ≥ 25) groups. Independent measures t-tests were used to compare participant characteristics by weight status and sex.

Appeal and desire were analyzed using repeated measures t-tests to study differences in ratings among food categories, and food appeal and desire to eat for each food category. Pearson correlations were used to analyze the relationships among appeal and desire by food category and participant characteristics. Participant characteristics of interest included: BMI, hunger, fullness and dietary restraint and disinhibition. To study the effect of portion size on appeal and desire ratings, difference scores were calculated between ratings for the large and small portions. Previous reports suggest differential responses to portion size by sex and weight status [35]. To account for this, weight status, sex, level of fullness and interactions where included in the repeated measures analyses of variance model when testing the effect of portion size on ratings. In the case of a significant interaction, least squared means were compared using a Tukey-Kramer adjustment for multiple comparisons (*p *< 0.05). The USDA Food Database was used for the dietary analyses of the energy (kcal/food (g)). Pearson correlational analyses were used to study the relationship between appeal and desire ratings and energy density. Pearson correlations and Cronbach's α were calculated to assess test/retest reliability utilizing the repeated images that were imbedded into the image set.

## Results

### Food Appeal & Desire to Eat Ratings

The overall mean ratings for appeal and desire are presented in Table 3. Food appeal was significantly higher than desire to eat (57.9 ± 11.6 v. 44.7 ± 18.0; t = 3.14; *p *< 0.05; Table 3), yet appeal and desire were positively correlated (*r *= 0.57, *p *< 0.001). When examining at the food categories, fruit had a mean appeal rating of 71.8, which was significantly higher than all other food categories (ratings ranged from 49.3 - 61.7). A similar pattern was observed in desire (Table 3).

**Table 3:** Ratings of images by food category

	Food Appeal (± SD)	Desire to Eat (± SD)
Fruit	71.8 (12.7)*^a^	59.7 (19.9)*^b^
Discretionary foods	61.7 (14.1)^a^	45.9 (21.6)^b^
Grains	58.1 (13.9)^a^	44.3 (21.0)^b^
Dairy	49.3 (17.8)^a^	38.3 (21.8)^b^
Vegetables	56.2 (13.2)^a^	41.5 (19.3)^b^
Mixed dishes	55.2 (14.9)^a^	42.6 (21.6)^b^
Protein	53.4 (17.8)^a^	40.9 (23.6)^b^
Total	57.9 (11.6)^a^	44.7 (18.0)^b^

*indicates fruit is rated significantly higher than the other food categories (*p *< 0.05)

^a, b ^different superscripts indicate significant differences between appeal and desire within a food category (*p *< 0.05)

BMI was positively associated with ratings for desire to eat, but not food appeal (Table 4). When examining the relationship between weight status and ratings by food category, BMI was the only significant correlate of appeal ratings for discretionary foods (Table 4). The relationship between BMI and desire to eat discretionary foods was driven by overweight individuals in that BMI was correlated with desire to eat discretionary foods in overweight (*r *= 0.38, *p *< 0.01), but not lean individuals. Desire to eat discretionary foods, grains, vegetables, and protein all had similar correlation with BMI, followed closely by trending relationship with mixed dishes and dairy foods. For ratings of desire to eat of the fruit category was the only category not significantly correlated (or trending towards significance) to BMI.

**Table 4:** Pearson correlations between appeal and desire ratings by food category and participant characteristics

	BMI	Hunger	Fullness	Dietary Restraint	Dietary Disinhibition
**Food Appeal**					

All foods	.15	.16	-.10	-.11	-.08
Fruits	.02	-.12	.12	.02	-.12
Discretionary foods	.29**	-.03	-.01	-.07	.03
Grains	.13	.15	-.14	-.10	-.04
Dairy	.01	.12	-.01	-.04	-.12
Vegetables	.10	.06	-.04	-.07	-.07
Mixed Dishes	.12	.30**	-.23*	-.14	-.01
Protein	.13	.29**	-.20*	-.16	-.07

**Desire to Eat**					

All Foods	.20*	.34**	-.24**	-.21*	.11
Fruits	.04	.06	-.01	-.11	.02
Discretionary foods	.20*	.09	-.07	-.15	.19*
Grains	.19*	.30**	-.25**	-.21*	.15
Dairy	.16^^^	.36**	-.24**	-.09	.08
Vegetables	.18*	.31**	-.22*	-.19*	.07
Mixed Dishes	.17^^^	.41**	-.32**	-.21*	.12
Protein	.19*	.41**	-.29**	-.24**	.05

**p < 0.01

*p < 0.05

^^^*p *= 0.05

Hunger and fullness were associated with desire in the directions one would anticipate: i.e., as hunger increased, desire increased and as fullness increased, desire decreased. However, neither hunger nor fullness were associated with food appeal (Table 4). When analyzed by weight status, the relationship between hunger and food appeal was significant for overweight (*r *= 0.27, *p *= 0.05), but not lean individuals (*p *= 0.37). Analyses of appeal ratings by food categories revealed that only mixed dishes and protein were associated with hunger and fullness, whereas most food categories (except fruits and discretionary foods) were related to desire (Table 4).

Reported dietary restraint was negatively correlated to desire to eat all foods, but not significantly related to food appeal, whereas disinhibition was not significantly associated with either of the ratings (Table 4). Overweight individuals reported higher restraint (9.6 ± 4.7 v. 7.9 ± 4.3; p < 0.05) and disinhibition (6.8 ± 3.9 v. 5.4 ± 3.4; p < 0.05) relative to lean individuals, although these differences are clinically marginal. The relationship between desire to eat and dietary restraint was significant in lean (*r *= -0.24, *p *< 0.05), but not overweight individuals. When analyzed by food category, restraint was negatively associated with desire grains, vegetables, mixed dishes and protein, but was not related to fruits, discretionary foods or dairy, notably both high fat food categories (Table 4). Similar to the findings with hunger and fullness, the two highest rated categories i.e., discretionary foods and fruits, were not associated with restraint. Disinhibition was positively correlated with desire to eat discretionary foods (Table 4).

### Portion Size, Energy Density & Reliability

Mean difference scores (large portion rating - small portion rating) revealed that individuals rated large portions higher than small portions for both appeal, where the large portion was rated 2.6 ± 4.4 higher than the small portion (*p *< 0.001), as well as desire to eat, where the large portion was rated 1.6 ± 3.9 higher than the small (*p *< 0.001). Analysis of variance main effects of weight status and portion size were observed for ratings of desire to eat: overweight participants' difference scores were significantly higher than lean individuals' scores for desire to eat (2.3 ± 0.5 v. 0.8 ± 0.5; *p *< 0.05). In addition, a significant weight status by sex interaction was observed, specifically overweight men's difference scores were larger compared to lean men's scores (2.7 ± 0.7 v. -0.3 ± 0.8; *p *< 0.05).

Pearson correlation analyses among scores for food appeal, desire, and energy and sugar densities of the foods were analyzed. Appeal and desire were negatively correlated with energy density of the foods presented in the images (*r *= -0.27, *p *< 0.01; *r *= -0.27, *p *< 0.01 respectively).

Measures of reliability for ratings of appeal and desire were high for both test-retest reliability and internal consistency: food appeal (*r *= 0.91, Cronbach's α = 0.95; *p *< 0.001) and desire to eat (*r *= 0.91, Cronbach's α = 0.95; *p *< 0.001).

## Discussion

Food appeal and desire to eat were correlated, but their differential relations with individual characteristics and different overall mean ratings indicate that participants discriminated between the two constructs despite using the same type of metric to measure them. The present results for appeal and desire to eat dovetail previous reports differentiating liking from wanting [17], and wanting's relation to weight status [19,36,37]. The consistency in these findings is encouraging given previous studies used different methods (i.e., button pushing vs., asking desire to eat) and terms (i.e., 'liking' v. 'food appeal'). This suggests these measures are capturing aspects of the same construct and confirm the ability for individuals to reliably differentiate between liking and wanting. However given the current lack of a gold standard, the ability to empirically test this notion is unavailable. Results from the present study suggest that the distinction between appeal and desire might be moderated by weight status. For example, BMI was positively related to desire, but not appeal ratings. This suggests that desire to eat a food item could play a larger role in the dysregulation of weight status relative to the food's preference. However, to date, the majority studies evaluating liking and wanting are cross-sectional and few involve food [17,37-39]. Therefore future studies should consider prospective designs to demonstrate the relations between liking and wanting, habitual intake and weight gain.

We observed that BMI was positively associated with desire to eat discretionary foods, however ratings for these foods had no association with hunger or fullness. This could suggest that overweight individuals' desire to eat highly palatable foods is "overriding" homeostatic mechanisms that control food intake (i.e., satiation). These results dovetail previous theories of the development of obesity where overweight individuals' eat for pleasure [19], consume energy dense foods in response to hedonic hunger [40], and are highly susceptible to environmental food cues [41].

The current data demonstrate that as lean individuals' restraint increases, their desire to eat a highly palatable food decreases. Overweight individuals' dietary restraint did not relate to their desire to eat a food, despite overweight individuals reporting slightly higher levels of dietary restraint. Notably, dietary restraint scores have been previously reported to be unrelated to measures of acute [42,43] or habitual intake [44,45] but were positively related to increases in weight [46,47] and onset of binge eating and bulimia [48,49]. Further, restraint has been previously reported to be positively associated with activity in reward-related brain regions when shown images of preferred foods [50] and when receiving a palatable food [51], suggesting the more restrained an individual is, the more pleasure they receive from seeing and/or consuming that food. In light of these findings it has been hypothesized that overweight individuals in particular can perceive themselves as 'restrained', but still habitually overeat [52]. Collectively, data from the present study support this notion, specifically that overweight individuals reported being restrained eaters, but that has no effect on their desire to consume food.

Contrary to our hypotheses we found that the fruit category was rated higher than all other food categories in both appeal and desire and we observed that energy density was inversely related to the ratings. Individuals are born with an innate preference for sweet [53] and thus it is possible that there is inborn predisposition for the higher ratings of fruit. It is also possible that the colorful nature of fruit could be responsible for its high rating. It is a possibility that fruit was rated higher than other food categories due to a response bias given fruit is generally perceived as healthy. However, if this notion held true across the food categories, one would anticipate that vegetables would also be rated higher. The seasonality of fruit could influence these ratings, because fruit's appearance, taste and cost vary by the time of year. However, a seasonality effect is unlikely given data collection occurred from late summer to mid-winter spanning multiple seasons.

Additional analyses by food categories revealed that only the two 'sweetest' tasting and highest rated food categories, (discretionary foods and fruits) were not associated with hunger or fullness. These findings suggest that discretionary foods and fruits might be foods commonly eaten outside of hunger. Eating despite feeling full can play a role in excess calorie consumption and weight regulation [54-57].

Hunger and fullness were associated with desire to eat, but not food appeal ratings. This could be a result of food appeal being a more stable trait-like characteristic whereas desire to eat could assess a particular state at that point in time specifically influenced by the satiating effects of the nutritional shake consumed prior to the ratings. Because participants were feeling full, they might want to consume a food less, but that food item is still appealing. Because fullness is a transient state and the participants were asked to rate their desire at that point in time, we suggest that individual's interpreted desire to eat as an immediate sensation (e.g., "I want this food right now"), whereas appeal was more of a generalized, stable feeling. Finlayson and colleagues reported differences in liking and wanting ratings when individuals were in differing energy states [16,17]. Therefore, we hypothesize that if our study were replicated in the fasted state, ratings for desire to eat would be more similar to food appeal. This hypothesis raises the question of whether appeal and desire originate in the same manner. Preference (similar to food appeal) is developed, in large part, via repeated exposure and physiological learning [58,59], but it is unclear how individuals develop desire (or wanting).

It is important to acknowledge limitations in the present investigation. First, there are multiple sensory inputs and feedback mechanisms responsible for eating behavior. This study specifically focused on the individual's response to the visual food cues while controlling for energy state, independent of smell and taste. Because the participants' did not actually taste the food, the results rely on their previous experiences with the presented foods; future studies ideally should measure responsivity to taste. However, this is a considerably more challenging study design when attempting to present and taste a large number of foods, which invoke effects of satiety. Further, visual food cues contribute to food selection and meal initiation and thus can be thought of as anticipatory cues to consumption. Food intake and weight regulation are complex processes and the present results should not be over generalized. Additionally, while there was support (that is, statistical significance) for our hypothesis that overweight individuals would rate larger portions higher than smaller portions, the effects were very small (2-3 points; scale range 0-100). Therefore, the public health significance and generalizability of these results is limited. While we have reported differential effects of portion size on intake by weight status [35] null effects have also emerged [5]. Lastly, the validity of using of VAS across group comparisons (e.g., lean vs. obese, male vs. female) has been questioned [60-62]. Specifically, the anchors used in VAS may denote systematically different perceived intensities to the different groups. The present results using across group comparisons should be interpreted with caution and future studies should consider the use of generalized labeled magnitude scale as described by Bartoshuk and colleagues (2004).

## Conclusions

This study has resulted in a reliable computer paradigm that can assess and differentiate between food appeal and desire to eat foods using a similar metric. This tool can prove useful given the ease of dissemination and flexibility in the number of foods tested without an impact on satiety. Results indicate that individual and food characteristics should also be considered when assessing the appeal and desire of food images. Future studies should address how these ratings relate to physiological measures (e.g., brain activation), food intake and longer-term weight regulation.

## Competing interests

The authors declare that they have no competing interests.

## Authors' contributions

KSB, MAC and SLJ conceived of the study and participated in its design and coordination. KSB collected and analyzed the data, drafted the manuscript and was responsible for incorporating the remaining authors' comments. SLJ assisted in the data analysis and drafting the manuscript. JI participated in the design of the study, and wrote the computer paradigm ImageRate. All authors provided feedback on drafts of the manuscript and read and approved the final manuscript.
